# Mitochondria-Targeted Antioxidant Mitoquinone Maintains Mitochondrial Homeostasis through the Sirt3-Dependent Pathway to Mitigate Oxidative Damage Caused by Renal Ischemia/Reperfusion

**DOI:** 10.1155/2022/2213503

**Published:** 2022-09-20

**Authors:** Hu Mao, Ye Zhang, Yufeng Xiong, Zijing Zhu, Lei Wang, Xiuheng Liu

**Affiliations:** ^1^Department of Urology, Renmin Hospital of Wuhan University, Wuhan, 430060 Hubei, China; ^2^Division of Nephrology, Renmin Hospital of Wuhan University, Wuhan, 430060 Hubei, China

## Abstract

Mitochondrial dysfunction is a critical factor contributing to oxidative stress and apoptosis in ischemia-reperfusion (I/R) diseases. Mitoquinone (MitoQ) is a mitochondria-targeted antioxidant whose potent anti-I/R injury capacity has been demonstrated in organs such as the heart and the intestine. In the present study, we explored the role of MitoQ in maintaining mitochondrial homeostasis and attenuating oxidative damage in renal I/R injury. We discovered that the decreased renal function and pathological damage caused by renal I/R injury were significantly ameliorated by MitoQ. MitoQ markedly reversed mitochondrial damage after I/R injury and inhibited renal reactive oxygen species production. In vitro, hypoxia/reoxygenation resulted in increased mitochondrial fission and decreased mitochondrial fusion in human renal tubular epithelial cells (HK-2), which were partially prevented by MitoQ. MitoQ treatment inhibited oxidative stress and reduced apoptosis in HK-2 cells by restoring mitochondrial membrane potential, promoting ATP production, and facilitating mitochondrial fusion. Deeply, renal I/R injury led to a decreased expression of sirtuin-3 (Sirt3), which was recovered by MitoQ. Moreover, the inhibition of Sirt3 partially eliminated the protective effect of MitoQ on mitochondria and increased oxidative damage. Overall, our data demonstrate a mitochondrial protective effect of MitoQ, which raises the possibility of MitoQ as a novel therapy for renal I/R.

## 1. Introduction

Acute kidney injury (AKI) is a common clinical condition characterized by rapid loss of renal function in a short period of time [1]. Renal ischemia/reperfusion (I/R) injury is one of the leading causes of acute kidney injury and is associated with high morbidity, hospitalization, and mortality [2]. Factors such as partial nephrectomy, trauma, hypotension, renal artery angioplasty, and sepsis are common triggers of renal I/R injury [3]. The pathophysiological process of renal I/R injury is very complex, involving oxidative stress, apoptosis, inflammatory infiltration, autophagy, and mitochondrial dysfunction [4]. Although some potential therapeutic strategies for renal I/R injury have been obtained through animal experiments, the specific mechanisms are still unknown. Hence it is important to investigate the molecular mechanisms underlying the progression of renal I/R injury and to explore potential therapeutic agents to reduce mortality due to AKI.

An increasing number of studies have shown that oxidative stress-mediated mitochondrial damage is a crucial reason for renal I/R injury [5]. During ischemia, the blood supply to the kidney is reduced or absent, and the cells are unable to obtain energy metabolites such as oxygen and glucose. Hypoxia depletes intracellular ATP, inactivates mitochondrial oxidative phosphorylation, and leads to compensatory conversion to anaerobic metabolism [6]. In contrast, during reperfusion, the re-entry of oxygen into ischemic tissue leads to reactivation of the mitochondrial respiratory chain and the production of massive amounts of free radicals, resulting in excess reactive oxygen species (ROS) and extensive oxidative damage [7]. The excess ROS further induces mitochondrial DNA damage and inhibits mitochondria-mediated ATP production, which can exacerbate the disruption of intracellular energy metabolism and promote cellular necrosis and apoptosis [8].

Numerous studies have shown that mitochondrial homeostasis is the key element of cell biological activity under physiological and pathological conditions. Mitochondrial homeostasis mainly includes mitochondrial biogenesis and mitochondrial dynamics [5]. Mitochondrial homeostasis is intimately regulated by mitochondrial dynamics, including mitochondrial fission, fusion, and autophagy [9, 10]. Abnormal mitochondrial fission induces inhibition of mitochondrial respiration, while mitochondrial fusion promotes ATP synthesis by facilitating communication between mitochondria, inhibiting mitochondrial breakage, and maintaining the integrity of mitochondrial DNA [11, 12].

Mitoquinone (MitoQ) is a mitochondria-targeted antioxidant. In contrast to conventional antioxidants, which have difficulty in entering the mitochondrial interior, MitoQ contains a lipophilic triphenylphosphonium cation (TPP) conjugated to coenzyme Q10, which results in rapid entry into the mitochondria and hundreds of times stronger ROS scavenging capacity [13, 14]. MitoQ has been shown to be effective in reducing mitochondrial damage and exerting powerful antioxidant capacity in a variety of diseases [15–17]. Moreover, a study preliminarily explored the protective role of MitoQ in renal I/R injury, but the underlying mechanisms have not been clarified [18]. Therefore, the potential intracellular mechanisms by which MitoQ exerts its antirenal I/R injury effects remain to be explored and elucidated.

Mammalian sirtuins (Sirt1-7) are a family of deacetylases that exert their activity mainly through nicotinamide adenine dinucleotides (NAD^+^), of which Sirt3 is mainly expressed in mitochondria [19]. The mitochondrial protective role of Sirt3 has been demonstrated in ischemic diseases such as cardiac I/R and intestinal I/R [10, 20]. Sirt3 regulates various cell biological activities through its deacetylation, including mitochondrial dynamics, energy metabolism, and cellular responses to oxidative stress [21]. Therefore, it is reasonable to assume that Sirt3 is essential for the maintenance of mitochondrial homeostasis.

In our study, the protective effects of MitoQ on renal ischemia/reperfusion injury have been verified by establishing in vivo and in vitro models, focusing on the substantial benefits of MitoQ in maintaining mitochondrial homeostasis and mitigating oxidative damage, and further elucidating the potential mechanism by which MitoQ exerts its protective effects may depend on the Sirt3 pathway.

## 2. Materials and Methods

### 2.1. Animals and Experimental Grouping

All procedures in this study were approved by the Wuhan University Laboratory Animal Committee (No.00012986), and all animal experiments were performed in accordance with the principles of Animal Care of Wuhan University (Wuhan, China). All procedures were also in accordance with the Guide for the Care and Use of Laboratory Animals published by the National Institutes of Health. Adult male C57BL/6 mice were purchased from the Animal Experimental Center of the First Clinical College of Wuhan University. All mice weighed 20-25 g and were 6-7 weeks of age. Mice were housed in a pathogen-free facility, maintained at appropriate temperature and humidity, adapted to a 12-hour light/dark cycle, and had free access to water and food.

42 mice were randomly divided into 7 groups (*n* = 6 per group): the Sham group; the Sham+MitoQ group; the I/R (ischemia 45 min, reperfusion 0 h, 12 h, and 24 h) group; the I/R+MitoQ group; and the I/R+MitoQ+3-TYP group. The Sham group received sham surgery; the Sham+MitoQ group received intraperitoneal injection of MitoQ (5 mg/kg) for 3 consecutive days before sham surgery; the I/R group underwent renal ischemia/reperfusion injury; the I/R+MitoQ group received intraperitoneal injection of MitoQ (5 mg/kg) for 3 consecutive days before renal ischemia/reperfusion; and the I/R+MitoQ+3-TYP group received intraperitoneal injection of MitoQ (5 mg/kg) and 3-TYP (50 mg/kg) for 3 consecutive days before renal ischemia/reperfusion. MitoQ (MedChemExpress, HPLC ≥98.00%, HY-100116A, USA) and 3-TYP (MedChemExpress, HPLC ≥99.93%, HY-108331, USA) were administered at concentrations referenced to previous studies [22, 23].

### 2.2. Animal I/R Model

The mice renal I/R model was performed as described previously [24]. Briefly, mice were anesthetized with sodium pentobarbital (50 mg/kg) and fixed on a constant temperature table to maintain body temperature. The mice were incised in the midline of the abdomen, and the right kidney was freed and removed. The left renal tip was then clamped closed using a noninvasive vascular clip for 45 min, and the noninvasive clip was then removed. Before suturing the abdomen, body fluid balance was maintained with a drop of PBS. Reperfusion was performed for 0 h, 12 h, and 24 h according to experimental needs. Only the right kidney was resected in the Sham and the Sham+MitoQ groups. After completion of reperfusion, mice were executed by intraperitoneal injection of sodium pentobarbital, and fresh blood and kidneys were collected immediately.

### 2.3. Renal Function Analysis

1 ml of fresh blood from mice was used to analyze serum creatinine (Cr) and urea nitrogen (BUN). Mice were assessed for renal function using the creatinine and urea commercial kit (Nanjing Jiancheng Bioengineering Institute, Nanjing, China) according to the manufacturer's instructions.

### 2.4. Histological Staining

Fresh kidney tissues were fixed in 4% paraformaldehyde and then embedded using paraffin. Sections of 4 *μ*m thickness were prepared and subsequently stained using a hematoxylin and eosin (H&E) kit (Beyotime Biotechnology, C0105S, China). Kidney sections were examined and scored by two renal pathologists who were unaware of the experiment to assess the degree of pathological damage. The scoring classified renal tubular injury (tubular necrosis, tubular dilatation, tubular pattern formation, and brush border absence) into five classes: 0, none; 1, <25%; 2, 25%-50%; 3, 50%-75%; and 4, >75%.

### 2.5. Immunohistochemistry

Paraffin sections of kidney tissues were dewaxed in xylene and then rehydrated in 100%, 90%, and 70% concentrations of alcohol. Immunohistochemical staining was performed using UltraSensitiveTM SP (mouse/rabbit) IHC Kit (MXB Biotechnologies, KIT-9710, China). Briefly, tissue sections were subjected to antigen repair, blocking nonspecific binding and blocking, followed by incubation of sections with Sirt3 (1: 200, Cell Signaling, 2627S), KIM-1 (1 : 100, Cell Signaling, 14971S), and Caspase-3 (1 : 1000, Cell Signaling, 9662S) antibodies were incubated overnight at 4°C. The secondary antibody was incubated at room temperature the next day and labeled with horseradish peroxidase. Subsequently, DAB was used for color development and the nuclei were restained with hematoxylin. At 400x magnification, randomly selected renal cortical fields were taken and photographed to assess staining intensity. The relative mean integrated optical density (IOD) of each group was determined using Image-Pro Plus (version 7.0 for Windows, Media Cybernetics, Rockville, Maryland USA) and divided by the mean IOD of the control to give the score for each group.

### 2.6. TdT-Mediated dUTP Nick-End Labeling Staining Analysis

Paraffin sections of kidney tissue were dewaxed and then rehydrated in a gradient concentration of alcohol. The TUNEL kit (Beyotime Biotechnology, C1090, China) was used to detect TUNEL (TdT-mediated dUTP Nick-End Labeling) positive cells. Briefly, proteinase K was incubated for 30 min at 37°C, washed with PBS followed by dropwise addition of TUNEL assay, and placed in a dark environment for 60 min. Nuclei were stained with DAPI before sealing the sections. Renal tissue sections were observed using a fluorescence microscope, and the average number of TUNEL-positive cells was calculated by randomly selecting the field of view.

### 2.7. Mitochondrial Morphology Analysis

For mice kidney tissues, transmission electron microscopy (TEM) was used for analysis. After harvesting mice kidney tissues, they were fixed using glutaraldehyde and made into electron microscopic specimens. Mitochondrial morphology was assessed using transmission electron microscopy with randomly selected fields of view according to standard procedures.

For HK-2 cells, Mito Tracker Red Staining analysis was performed. HK-2 cells were spread on cell crawlers, and after treatment was completed, the cells were incubated with Mito Tracker Red CMXRos (Beyotime Biotechnology, C1035, China) for 25 min at 37°C in the dark, washed with PBS, and the cell crawlers were removed and stained with DAPI before sealing. Mitochondrial morphology was subsequently observed by fluorescence microscopy.

### 2.8. Cell Culture and Hypoxia-Reoxygenation (H/R) Model

The human renal proximal tubular epithelial cell line (HK-2), from the American Typical Culture Collection (ATCC, Rockville, MD, United States), is a human renal epithelial cell line derived from the cortical proximal tubule. HK-2 cells were cultured in a DMEM medium containing 10% fetal bovine serum (FBS) in an incubator set to 37°C, 5% CO_2_, and 95% air conditions. A cellular hypoxia-reoxygenation (H/R) model was established as described previously [24]. Briefly, HK-2 cells were cultured in glucose- and serum-free medium and placed in hypoxic conditions (1% O_2_, 94% N_2_, and 5% CO_2_) for 12 h. After completion of hypoxia, the medium containing glucose and serum was replaced and placed in normoxic (5% CO_2_ and 95% air) conditions for 0 h, 3 h, and 6 h. For drug treatment, the cells were incubated before hypoxia using MitoQ (0.1 *μ*M, 0.5 *μ*M) was pretreated for 24 h.

### 2.9. Western Blot Analysis

Protein expression levels of Sirt3, Drp1, Mfn2, SOD1, and SOD2 were detected in kidney tissues and HK-2 cells according to standard protocols. GAPDH was used as an internal reference. Briefly, after lysis of cells and tissues using RIPA to obtain total protein, protein concentrations were detected using the BCA protein assay kit (Beyotime Biotechnology, P0006, China). Protein samples were separated by polyacrylamide gel electrophoresis and transferred to PVDF membranes. The membranes were closed in 5% skimmed milk powder at room temperature for 2 h, and then mixed with the membrane against Sirt3 (1 : 1000, Cell Signaling, 2627S), Drp1 (1 : 500, Santa Cruz Biotechnology, sc-271583), Mfn2 (1 : 500, Santa Cruz Biotechnology, sc-515647), SOD1 (1 : 1000, Proteintech North America, 10269-1-AP), SOD2 (1 : 5000, Proteintech North America, 24127-1-AP), and GAPDH (1: 5000, Proteintech North America, 10494-1-AP) antibodies at 4°C overnight. The next day the antibodies were washed with TBST and incubated with secondary antibodies for 60 min at room temperature. Western blots were evaluated using an enhanced chemiluminescence solution (Thermo Fisher Scientific, Waltham, MA, USA). Data analysis was performed using Image-Pro Plus (version 7.0 for Windows, Media Cybernetics, Rockville, Maryland USA) to quantify protein levels.

### 2.10. Silencing Sirt3 by siRNA Transfection

Sirt3 (si-Sirt3; GenePharma, Shanghai, China) or negative control- (si-NC; Santa Cruz Biotechnology, Santa Cruz, CA, USA) specific small interfering RNA (siRNA) was transfected into HK-2 cells. The efficiency of silencing Sirt3 after transfection was determined by western blotting experiments.

### 2.11. Immunofluorescence Chemistry

HK-2 cells were spread on cell crawl sheets, and after completing treatment according to experimental needs, they were fixed with 4% paraformaldehyde for 15 min, followed by permeabilization with 0.5% TritonX-100 at room temperature for 10 min, and then closed with 10% goat serum at room temperature for 60 min.

Incubate with the antibody against Sirt3 (1 : 50, Santa Cruz Biotechnology, sc-365175) overnight at 4°C. The next day, incubate with the secondary antibody at room temperature and in the dark for 60 min. Stain the nuclei with DAPI before sealing the slices and subsequently observe the fluorescence intensity using a fluorescence microscope.

### 2.12. Reactive Oxygen Species (ROS) Assay

For kidney tissues, reactive oxygen species levels in the kidney were measured using the Dihydroethidium kit (Beyotime Biotechnology, S0063, China). Briefly, mice kidney frozen sections were incubated with Dihydroethidium (DHE) for 30 min at 37°C in the dark, and nuclei were stained with DAPI before sealing the sections. For HK-2 cells, ROS levels in HK-2 cells were assessed using a reactive oxygen species detection kit (Beyotime Biotechnology, S0033S, China). HK-2 cells were stained with Hoechst 33342 (Beyotime Biotechnology, C1027, China), and dichlorodihydrofluorescein diacetate (DCFH-DA) were incubated together for 20 min at 37°C in the dark. The fluorescence intensity was observed using a fluorescence microscope with a randomly selected field of view.

### 2.13. Mitochondrial Membrane Potential (MMP) Assay

According to the instructions of the mitochondrial membrane potential assay kit (JC-1, Beyotime Biotechnology, C2006, China), HK-2 cells were incubated with JC-1 staining solution and Hoechst 33342 (Beyotime Biotechnology, C1027, China) for 20 min at 37°C in the dark. The green and red fluorescence intensity was observed using fluorescence microscopy after PBS washing.

### 2.14. ATP Concentration Assay

ATP assay kit (Beyotime Biotechnology, S0026, China) was used to detect the ATP content of HK-2 cells. After lysing the cells by adding lysis solution according to the instructions, the cells were centrifuged at 12,000 g for 5 min at 4°C, and the supernatant was collected. The samples were mixed with the ATP assay working solution, and the optical density values were detected by SpectraMax Paradigm multimode enzyme marker (molecular devices).

### 2.15. Flow Cytometric Analysis

HK-2 cells were collected and the apoptosis rate was detected using the apoptosis detection kit annexin V-FITC/PI apoptosis kit (Multi Sciences Biotech Co., Ltd., 70-AP101-100, China). Briefly, approximately 100,000 HK-2 cells (including cells in the culture medium) were collected by centrifugation, binding buffer, FITC, and PI were added, and apoptosis was assessed using CytoFLEX (Beckman Coulter Biotechnology (Suzhou) Co., Ltd., China) after incubation was completed at room temperature.

### 2.16. Statistical Analysis of Data

All data are expressed as mean ± standard error of the mean (SEM). Differences were compared by one-way analysis of variance (ANOVA) and unpaired student's *t*-test. One-way ANOVA was used to test the homogeneity of variance of the data and *t*-test was used to test the difference between the two groups. Differences at *p* < 0.05 were considered statistically significant. All data were statistically analyzed using GraphPad Prism (version 8.0.2 for Windows, GraphPad Software, San Diego, California USA).

## 3. Results

### 3.1. MitoQ Ameliorated Renal Dysfunction, Pathological Injury, and Apoptosis after Renal I/R in Mice

**Figure 1:**
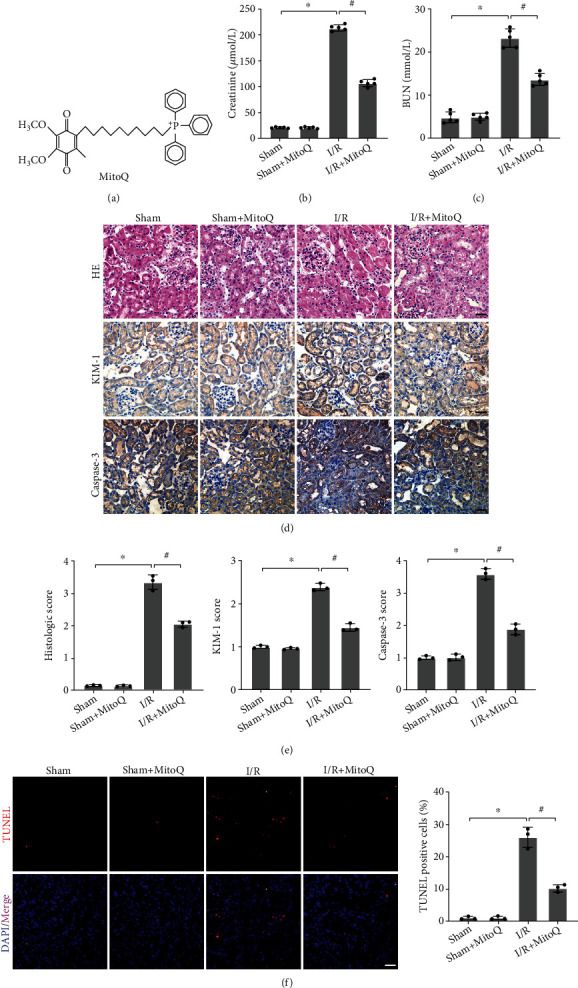
MitoQ ameliorated renal I/R injury in mice. (a) The chemical structure diagram of MitoQ. (b and c) Mice renal function assays including serum creatinine (Cr) and urea nitrogen (BUN). (d and e) Representative images of H&E staining and immunohistochemistry of KIM-1 and Caspase-3 in mice kidney tissues (×400, scale bar = 20 *μ*m) and related quantitative analysis. (f) TUNEL assay to assess the level of apoptosis in kidney cells and their quantitative analysis (×400, scale bar = 20 *μ*m). Values are expressed as mean ± SEM. *n* = 3 − 5. ^∗^*p* < 0.05 compared with the Sham group, ^#^*p* < 0.05 compared with the I/R group.

In this experiment, we established a mice renal I/R injury model to verify the protective effect of MitoQ (Figure 1(a)) in vivo. When mice experienced renal I/R injury, the levels of serum Cr and BUN were substantially increased, which represented severe renal dysfunction. However, intraperitoneal injection of MitoQ before ischemia significantly reduced the levels of serum Cr and BUN (by 50% and 41%, respectively) (Figures 1(b) and 1(c)). We further examined the renal pathological damage by H&E staining (Figures 1(d) and 1(e)). The morphology of the kidney sections in the Sham group was normal and the brush border membrane was well-preserved, whereas the renal tubular damage was evident in the IR group, as evidenced by tubular cell swelling, nuclear consolidation, and tubular lumen dilation and brush border detachment. As expected, MitoQ pretreatment significantly reduced renal pathological damage and preserved tubular structures and brush borders. Consistent with the H&E staining results, immunohistochemistry revealed high expression of kidney injury molecule-1 (KIM-1), a sensitive marker of renal tubular injury, in the I/R group, whereas KIM-1 expression was reduced in the kidneys of MitoQ pretreated mice (Figures 1(d) and 1(e)). Furthermore, we examined the effect of MitoQ on apoptosis in kidney cells. Immunohistochemical results showed that MitoQ partially abrogated the elevated expression of Caspase-3 (a marker of apoptosis) caused by I/R (Figures 1(d) and 1(e)). Similarly, TUNEL assays showed that MitoQ significantly reduced the number of TUNEL-positive cells (Figure 1(f)). Altogether, these data suggest that MitoQ can exert a remarkable renoprotective effect after renal I/R injury in mice.

### 3.2. MitoQ Attenuated Mitochondrial Damage, Promotes Mitochondrial Fusion, and Inhibits Oxidative Stress in Renal Tissue

**Figure 2:**
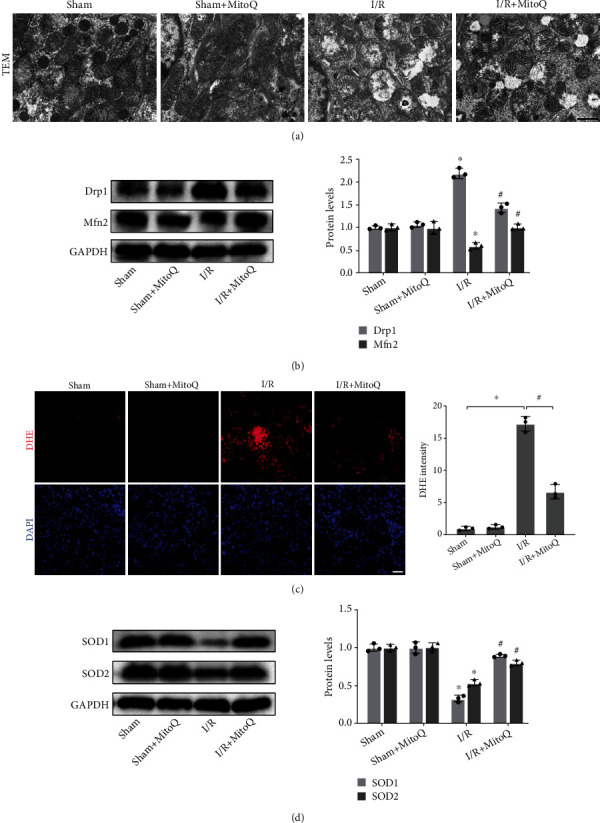
MitoQ attenuated mitochondrial damage and inhibited renal oxidative stress after renal I/R in mice. (a) Transmission electron microscopy (TEM) observation of mitochondrial morphology in renal tubular epithelial cells (×8000, scale bar = 1 *μ*m). (b) Western blot detection of Drp1 and Mfn2 protein expression, quantitative analysis expressed as the relative level with the Sham group. (c) Dihydroethidium (DHE) staining to assess renal oxidation levels and their quantitative analysis (×400, scale bar = 20 *μ*m). (d) Western blot detection of SOD1 and SOD2 protein expression, quantitative analysis expressed as the relative level with the Sham group. Values are expressed as mean ± SEM. *n* = 3. ^∗^*p* < 0.05 compared with the Sham group, ^#^*p* < 0.05 compared with the I/R group.

Since MitoQ is a mitochondria-targeted antioxidant, we further explored whether MitoQ exerts renoprotective effects by protecting mitochondria. We observed the morphology of mitochondria in renal tubular epithelial cells by transmission electron microscopy (TEM), and we found that the mitochondria in the Sham group were morphologically normal, whereas the mitochondria in the I/R group were severely damaged, showing swollen and enlarged mitochondria, broken mitochondrial cristae, and formation of vacuoles. The changes in mitochondrial morphology after I/R were significantly reversed by MitoQ (Figure 2(a)). Mitochondrial fission and fusion are key factors in maintaining mitochondrial dynamics. We found that renal I/R injury promoted mitochondrial fission and reduced mitochondrial fusion, as evidenced by elevated expression of the mitochondrial fission-related protein Drp1 and decreased expression of the mitochondrial fusion protein Mfn2. MitoQ, in turn, promoted mitochondrial fusion by downregulating Drp1 and upregulating Mfn2 (Figure 2(b)).

Mitochondrial damage is currently considered to be the underlying cause of oxidative stress. High fluorescence intensity of Dihydroethidium (DHE) in post-I/R kidney tissues represents high levels of ROS, while MitoQ significantly reduced DHE levels (Figure 2(c)). Moreover, we detected the protein levels of two antioxidant enzymes, including superoxide dismutase 1 (SOD1) and superoxide dismutase 2 (SOD2), by western blotting. After I/R occurred, the protein expression of SOD1 and SOD2 were significantly downregulated and the intracellular antioxidant enzyme system was inactivated, while MitoQ could restore the protein expression levels of SOD1 and SOD2 and rescue the antioxidant enzyme system, thus scavenging the excess ROS (Figure 2(d)).

### 3.3. In Vitro, MitoQ Maintained Mitochondrial Function and Mitochondrial Morphology in HK-2 Cells after H/R

**Figure 3:**
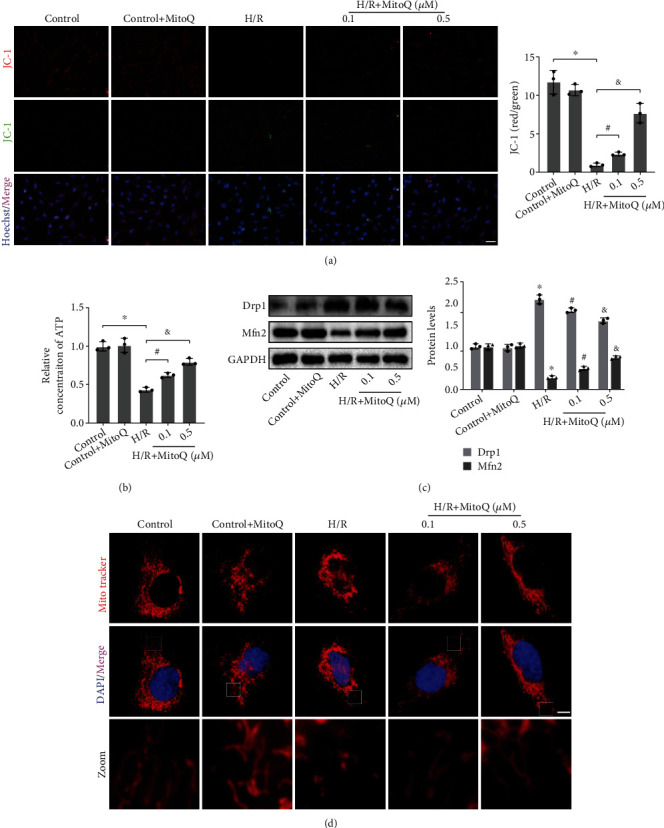
MitoQ restored mitochondrial homeostasis after H/R in HK-2 cells. (a) JC-1 assay of mitochondrial membrane potential in HK-2 cells (×200, scale bar = 50 *μ*m), quantitative analysis expressed as the ratio of JC-1 aggregates (red fluorescence) to JC-1 monomers (green fluorescence). (b) Relative ATP concentration in HK-2 cells. (c) Western blot detection of Drp1 and Mfn2 protein expression, quantitative analysis expressed as the relative level with the control group. (d) Mitochondrial morphology of Mito Tracker Red CMXRos-labeled HK-2 cells (×1000, scale bar = 5 *μ*m). Values are expressed as mean ± SEM. *n* = 3. ^∗^*p* < 0.05 compared with the control group, ^#^*p* < 0.05 and ^&^*p* < 0.05 compared with the H/R group.

Subsequently, we established a hypoxia/reoxygenation (H/R) model of human renal proximal tubular epithelial cells (HK-2) to verify the protective effect of MitoQ in vitro. H/R resulted in a significant decrease in mitochondrial membrane potential (MMP) in HK-2 cells, as evidenced by a decrease in JC-1 aggregates (red fluorescence) and an increase in JC-1 monomers (green fluorescence) (Figure 3(a)). And the ATP concentration required for cellular physiological activities was also markedly reduced (Figure 3(b)). However, the decreases in both mitochondrial membrane potential and ATP were dramatically reversed by MitoQ. Consistent with the in vivo results, H/R resulted in upregulation of Drp1 and downregulation of Mfn2 in HK-2 cells, which was prevented by MitoQ (Figure 3(c)). Additionally, by labeling mitochondria with Mito Tracker Red CMXRos, we found that mitochondria in the control group had normal morphology and showed continuous long strips, but mitochondria in the H/R group were broken and showed dot-like structures. MitoQ could partially restore mitochondrial morphology to normal (Figure 3(d)). These results together suggest that MitoQ exerts a mitochondrial protective effect in vitro.

### 3.4. MitoQ Suppressed Oxidative Stress and Apoptosis in HK-2 Cells after H/R

**Figure 4:**
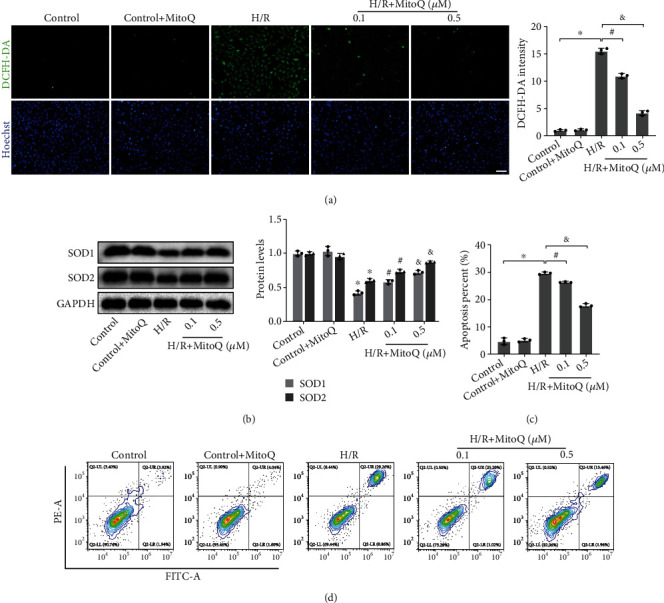
MitoQ inhibited oxidative stress and apoptosis in HK-2 cells. (a) Dichlorodihydrofluorescein diacetate (DCFH-DA) assessment of reactive oxygen species levels in HK-2 cells and their quantitative analysis (×100, scale bar = 100 *μ*m). (b) Western blot detection of SOD1 and SOD2 protein expression, quantitative analysis expressed as the relative level with the control group. (c and d) Flow cytometry detection of HK-2 cell apoptosis rate and their quantitative analysis. Values are expressed as mean ± SEM. *n* = 3. ^∗^*p* < 0.05 compared with the control group, ^#^*p* < 0.05 and ^&^*p* < 0.05 compared with the H/R group.

H/R caused oxidative stress in HK-2 cells. Compared with the control group, when HK-2 cells were exposed to H/R, intracellular ROS levels increased dramatically and protein expression of SOD1 and SOD2 was inhibited, resulting in the inactivation of the antioxidant enzyme system. However, MitoQ significantly reduced ROS levels and upregulated protein expression of SOD1 and SOD2, thereby inhibiting oxidative stress (Figures 4(a) and 4(b)). Detecting apoptosis by flow cytometry, we found that H/R caused apoptosis in about 30% of HK-2 cells, while MitoQ exerted a significant antiapoptotic effect, with a concentration of 0.5 *μ*M MitoQ reducing the rate of apoptosis to about 18% (Figures 4(c) and 4(d)).

### 3.5. MitoQ Significantly Upregulated the Expression Level of Sirt3 Protein after I/R and H/R

**Figure 5:**
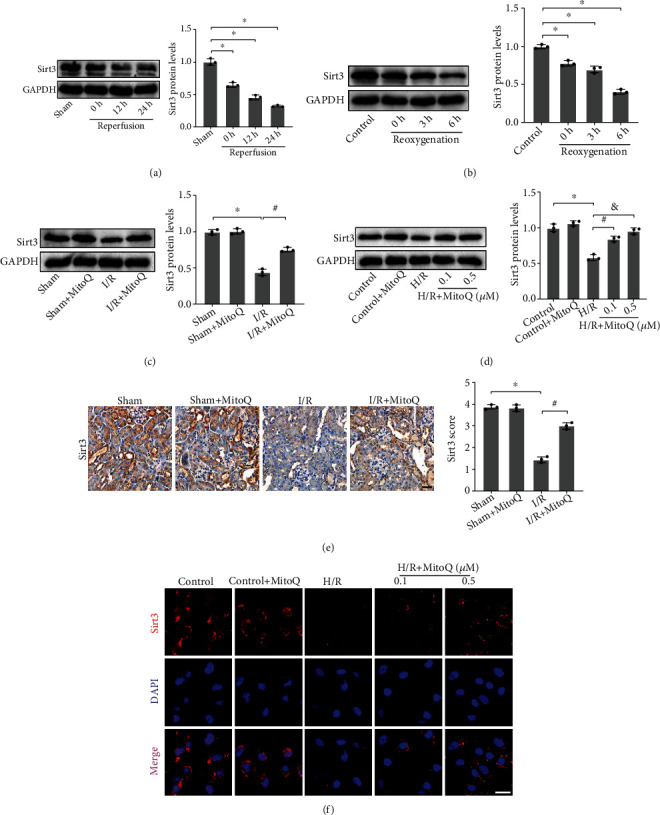
MitoQ significantly recovered the underexpression of Sirt3 resulted from I/R or H/R. (a and b) Western blotting to detect the effect of different reperfusion time or different reoxygenation time on the protein expression of Sirt3 and related quantitative analysis. (c and d) Western blotting to detect the effect of MitoQ on the protein expression of Sirt3, and related quantitative analysis. (e) Representative immunohistochemical pictures of Sirt3 in mice kidney tissue (×400, scale bar = 20 *μ*m) and related quantitative analysis. (f) Representative images of immunofluorescence of Sirt3 in HK-2 cells (×400, scale bar = 20 *μ*m). Values are expressed as mean ± SEM. *n* = 3. ^∗^*p* < 0.05 compared with the Sham or the control group, ^#^*p* < 0.05 and ^&^*p* < 0.05 compared with the I/R or the H/R group.

We then explored whether the role of MitoQ was related to the Sirt3 pathway. Firstly, we found that the protein expression of Sirt3 in mice kidneys gradually decreased with the prolongation of reperfusion time, and the protein expression of Sirt3 dropped to the lowest at the reperfusion time of 24 h (Figure 5(a)). Interestingly, the decrease in Sirt3 protein expression was significantly reversed by MitoQ (Figure 5(c)). The immunohistochemical results of Sirt3 were also consistent with the protein blotting results (Figure 5(e)).

Furthermore, consistent with in vivo, H/R resulted in a decrease of Sirt3 protein in HK-2 cells. The protein expression of Sirt3 was lowest at the time of reoxygenation up to 6 h (Figure 5(b)). MitoQ partially restored the expression level of Sirt3 compared to the H/R group, and the effect of MitoQ at a concentration of 0.5 *μ*M was more pronounced (Figure 5(d)). Immunofluorescence of HK-2 cells showed that H/R resulted in a noticeable decrease in the red fluorescence of Sirt3, while MitoQ markedly increased the red fluorescence intensity of Sirt3 protein (Figure 5(f)).

### 3.6. Silencing of Sirt3 Reversed the Protective Effect of MitoQ on Mitochondria of HK-2 Cells

**Figure 6:**
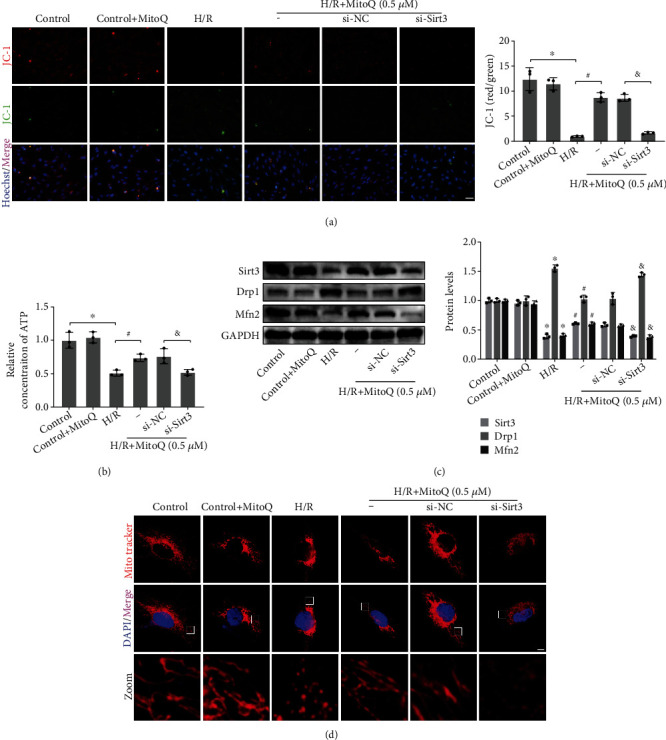
si-Sirt3 partially eliminated the mitochondrial protective effect of MitoQ on HK-2 cells. (a) JC-1 assay of mitochondrial membrane potential in HK-2 cells (×200, scale bar = 50 *μ*m), quantitative analysis expressed as the ratio of JC-1 aggregates (red fluorescence) to JC-1 monomers (green fluorescence). (b) Relative ATP concentration in HK-2 cells. (c) Western blot detection of Sirt3, Drp1, and Mfn2 protein expression, quantitative analysis expressed as the relative level with the control group. (d), Mitochondrial morphology of Mito Tracker Red CMXRos-labeled HK-2 cells (×1000, scale bar = 5 *μ*m). Values are expressed as mean ± SEM. *n* = 3. ^∗^*p* < 0.05 compared with the control group, ^#^*p* < 0.05 compared with the H/R group, and ^&^*p* < 0.05 compared with the H/R+MitoQ+si-NC group.

By silencing Sirt3 through small interfering RNA transfection of HK-2 cells, we further verified the dependence of MitoQ on the Sirt3 pathway. Compared with si-NC, si-Sirt3 significantly reversed the upregulation of Sirt3 protein expression caused by MitoQ (Figure 6(c)). Concomitantly, silencing of Sirt3 resulted in an increase in JC-1 monomer (green fluorescence) and a decrease in JC-1 polymer (red fluorescence). The ability of MitoQ to restore mitochondrial membrane potential (MMP) and promote ATP synthesis was abolished by silencing Sirt3 (Figures 6(a) and 6(b)). The alteration of mitochondrial dynamics-related proteins Drp1 and Mfn2 protein expression by MitoQ was also reversed by si-Sirt3 (Figure 6(c)). Besides, si-Sirt3 caused more mitochondrial breaks and promoted mitochondrial fission compared with the si-NC group of long-striped mitochondria (Figure 6(d)). Overall, our results suggest that the capability of MitoQ to maintain mitochondrial homeostasis in HK-2 cells is partially dependent on Sirt3.

### 3.7. Silencing Sirt3 Increased Oxidation Levels and Promoted Apoptosis in HK-2 Cells

**Figure 7:**
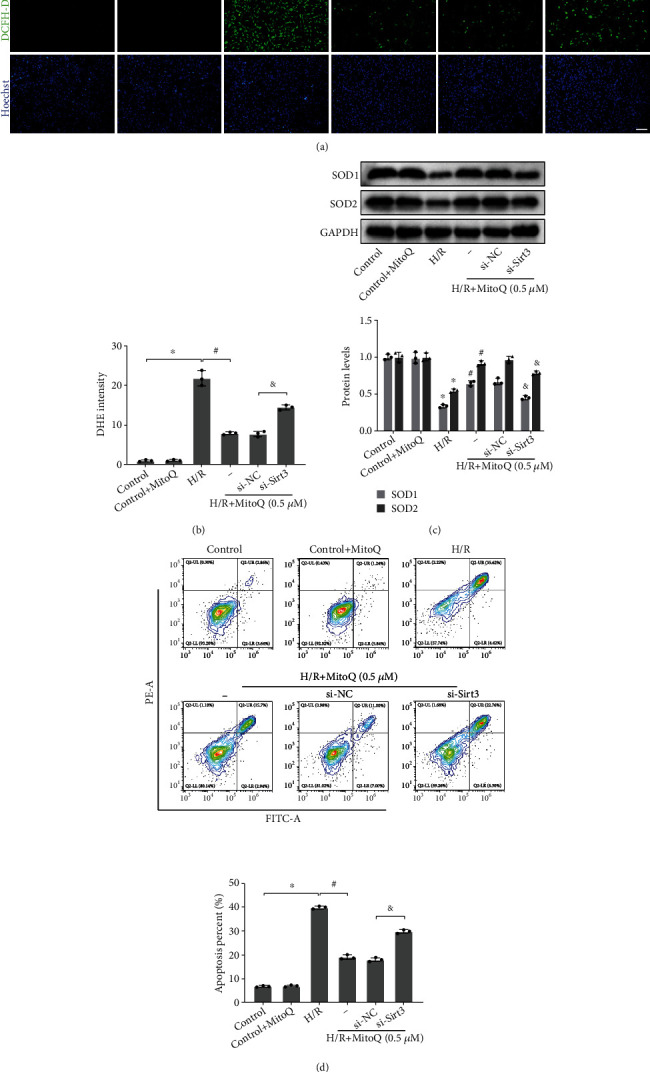
si-Sirt3 fractionally abrogated the ability of MitoQ to attenuate oxidative damage in HK-2 cells. (a and b) Dichlorodihydrofluorescein diacetate (DCFH-DA) assessment of reactive oxygen species levels in HK-2 cells and their quantitative analysis (×100, scale bar = 100 *μ*m). (c) Western blot detection of SOD1 and SOD2 protein expression, quantitative analysis expressed as the relative level with the control group.(d) Flow cytometry assay of apoptosis rate in HK-2 cells and their quantitative analysis. Values are expressed as mean ± SEM. *n* = 3. ^∗^*p* < 0.05 compared with the control group, ^#^*p* < 0.05 compared with the H/R group, and ^&^*p* < 0.05 compared with the H/R+MitoQ+si-NC group.

We then further explored the effect of silencing Sirt3 on MitoQ to attenuate oxidative damage in HK-2 cells. The excess ROS generated by HK-2 cells after undergoing H/R were efficiently scavenged by MitoQ, but this ability of MitoQ to scavenge ROS was partially eliminated by si-Sirt3 (Figures 7(a) and 7(b)). Moreover, si-Sirt3 significantly reduced the protein expression levels of the antioxidant enzymes SOD1 and SOD2 restored by MitoQ (Figure 7(c)). In terms of apoptosis, the antiapoptotic effect of MitoQ was also greatly reversed by si-Sirt3 (Figure 7(d)). These results suggest that the ability of MitoQ to attenuate oxidative stress and apoptosis in HK-2 cells requires the activation of Sirt3.

### 3.8. In Vivo, 3-TYP Eliminated the Ability of MitoQ to Attenuate Oxidative Stress and Apoptosis

**Figure 8:**
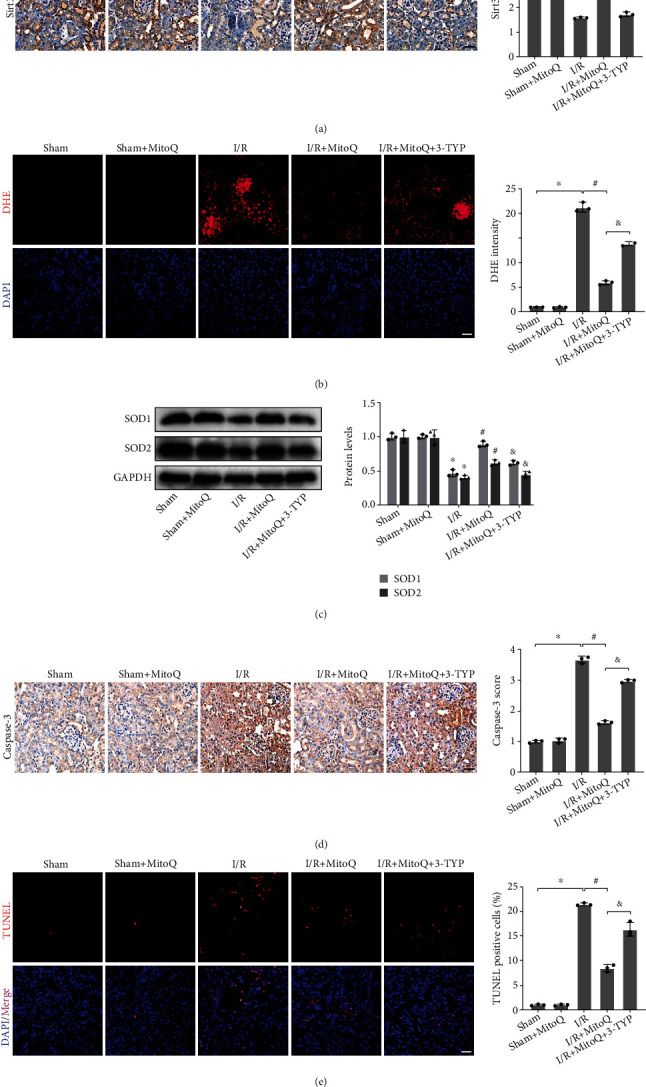
The Sirt3 selective inhibitor, 3-TYP, reversed the effect of MitoQ on inhibition of oxidative stress and apoptosis in mice kidney. (a) Immunohistochemical representative images of Sirt3 protein in mice kidney tissue (×400, scale bar = 20 *μ*m) and their quantitative analysis. (b) Dihydroethidium (DHE) staining to assess kidney oxidation levels (×400, scale bar = 20 *μ*m) and their quantitative analysis. (c) Western blot detection of SOD1 and SOD2 protein expression, quantitative analysis expressed as the relative level with the Sham group. (d) Immunohistochemical representative images of Caspase-3 protein in mice kidney tissue (×400, scale bar = 20 *μ*m) and their quantitative analysis. (e) TUNEL assay to assess renal apoptosis levels (×400, scale bar = 20 *μ*m) and their quantitative analysis. Values are expressed as mean ± SEM. *n* = 3. ^∗^*p* < 0.05 compared with the Sham group, ^#^*p* < 0.05 compared with the I/R group, and ^&^*p* < 0.05 compared with the I/R+MitoQ group.

In vivo, the selective inhibitor of Sirt3, 3-TYP, efficiently inhibited the high expression of Sirt3 protein caused by MitoQ by intraperitoneal injection (Figure 8(a)). ROS accumulated in kidney tissue after I/R injury in mice was potentially inhibited by MitoQ, but coadministration of 3-TYP and MitoQ promoted the production of ROS. MitoQ upregulated the protein expression of SOD1 and SOD2 after I/R and rescued the antioxidant enzyme system, but the function of the antioxidant enzyme system was partially inhibited by 3-TYP (Figure 8(b)). Furthermore, immunohistochemical results of the apoptosis marker Caspase-3 indicated that 3-TYP blocked the antiapoptotic effect of MitoQ after I/R. TUNEL assays also confirmed that 3-TYP increased the number of TUNEL-positive cells. Thus, the capacity of MitoQ to protect mice from renal I/R injury is partially reliant on the Sirt3 pathway.

## 4. Discussion

In the present study, we described a novel role of MitoQ in maintaining mitochondrial homeostasis and attenuating oxidative damage after renal I/R injury. In vivo, renal dysfunction, pathological injury, and apoptosis caused by renal I/R were prevented by MitoQ, which reduced mitochondrial fission and promoted mitochondrial fusion, thereby maintaining mitochondrial integrity and inhibiting oxidative stress. In vitro, the mitochondrial membrane potential and ATP concentration of HK-2 cells were restored by MitoQ, and mitochondrial dynamics and mitochondrial morphology were improved by MitoQ. Furthermore, MitoQ effectively inhibited oxidative stress and apoptosis in HK-2 cells. Through intensive study, we found that the potential mechanism of the protective effect of MitoQ was closely related to Sirt3. The protein expression level of Sirt3 was greatly upregulated by MitoQ after I/R and H/R. We further found that inhibition of Sirt3 partially blocked the protective effect of MitoQ by suppression of Sirt3 in vivo and silencing of Sirt3 in vitro. Together, these results thus suggest that MitoQ attenuates oxidative damage by maintaining mitochondrial homeostasis in renal I/R injury and that this protective effect of MitoQ is partly dependent on the activation of the Sirt3 pathway.

Excess ROS generated by mitochondrial dysfunction is a critical reason for the oxidative damage caused by I/R [25]. Ischemia leads to reduced efficiency of electron transport and disturbed energy metabolism in renal tubular epithelial and endothelial cells. Mitochondrial production of ROS alters the cellular redox potential thus leading to apoptosis as a hallmark event of I/R injury [7]. During reperfusion, the re-entry of oxygen alters the function of mitochondrial complex I and complex III, leading to electron leakage from mitochondria and secondary production of large amounts of ROS [26, 27]. Additionally, I/R damage leads to the inactivation of the antioxidant enzyme system and the inability of superoxide dismutase SOD1 and SOD2 to function to scavenge excess ROS [28, 29]. The accumulation of intracellular ROS and other oxygen radicals continues to cause damage to DNA and proteins, which can cause cytochrome c to leak from the mitochondria into the cytoplasm, thereby activating cystathionase to induce apoptosis [30]. Since damaged mitochondria become a source of oxidative stress, it is particularly important to supplement them with exogenous antioxidants to protect them. MitoQ is one of the most widely studied mitochondria-targeted antioxidants, which can rapidly enter cells and accumulate in mitochondria, where it is converted into the active antioxidant panthenol form to participate in the antioxidant process [31]. Recent studies have confirmed that MitoQ plays an excellent protective role in ischemic diseases such as retinal ischemia-reperfusion, renal transplantation, and intestinal ischemia-reperfusion [32–34]. In the present study, we found that MitoQ significantly ameliorated renal dysfunction in I/R mice, which is consistent with an earlier study [18]. Besides, the renal pathological damage, oxidative stress, and apoptosis caused by I/R injury were reversed by MitoQ as well.

The biological activities of cells are closely related to mitochondrial homeostasis, including metabolic reprogramming, apoptosis, and cell necrosis [35]. Abnormalities in mitochondrial dynamics cause disturbances in mitochondrial homeostasis. Studies have shown that mitochondrial damage caused by the imbalance of mitochondrial fission and fusion is a key cause of renal tubular injury [36]. Excessive mitochondrial fission leads to the accumulation of abnormal mitochondria, while mitochondrial fusion maintains a healthy mitochondrial network [10, 35]. In contrast to mitochondrial fission, mitochondrial fusion improves mitochondrial quality although it decreases the number of mitochondria. Mitochondrial fusion plays a protective role in maintaining mitochondrial function and morphology in a variety of diseases [37, 38]. The dynamin-related protein 1 (Drp1) and its receptor are the main proteins that regulate mitochondrial fission [39]. A study found that specific knockdown of proximal tubular Drp1 prevented mitochondrial fracture and thus reduced renal I/R injury [40]. Mitochondrial fusion-related protein 1/2 (Mfn1/2) is a kinetically related GTPase located on outer membrane mitochondria that mediate mitochondrial fusion by tethering the outer mitochondrial membrane [5]. In this study, we observed in vivo and in vitro that I/R or H/R damage resulted in upregulation of Drp1 and downregulation of Mfn2, while MitoQ prevented the changes in Drp1 and Mfn2 protein expression, thereby reducing mitochondrial fission and promoting fusion. Moreover, MitoQ was found to significantly reverse mitochondrial swelling and cristae breakage by transmission electron microscopy (TEM). In HK-2 cells exposed to H/R, mitochondrial membrane potential and ATP content were partially restored by MitoQ, and MitoQ treatment effectively reduced mitochondrial breaks. Together, these results suggest that MitoQ maintains mitochondrial homeostasis by balancing mitochondrial dynamics and restoring mitochondrial morphology and function.

Interestingly, our study revealed that the mechanism by which MitoQ exerts its action may be related to the activation of the Sirt3 pathway. Mitochondrial deacetylases include Sirt3, Sirt4, and Sirt5, which together mediate the post-translational modification of lysine residues, with Sirt3 playing a major role in regulating mitochondrial function [20, 41]. Several studies have reported that Sirt3 is a pivotal link in mitochondrial homeostasis and ROS management [42, 43]. Sirt3 also has a protective role in I/R injury. An investigation found that mice with Sirt3 knockdown were more susceptible to I/R injury and had reduced mitochondrial function [44]. Also, overexpression of Sirt3 enhanced mitochondrial fusion and improved renal dysfunction after I/R injury [45]. Consistently, our study found that Sirt3 expression was remarkably reduced after renal I/R injury, while MitoQ significantly restored Sirt3 expression both in vivo and in vitro. Silencing of Sirt3 abrogated the improvement of mitochondrial function and mitochondrial dynamics in HK-2 cells by MitoQ and promoted oxidative damage. Similarly, inhibition of Sirt3 in vivo reversed the function of MitoQ to attenuate oxidative stress and apoptosis caused by I/R injury. This suggests that the role of MitoQ in maintaining mitochondrial homeostasis to attenuate renal I/R oxidative injury appears to be dependent on the activation of the Sirt3 pathway.

## 5. Conclusions

Our study is the first to reveal the role of the MitoQ, a mitochondria-targeted antioxidant, in maintaining mitochondrial homeostasis and inhibiting oxidative stress in renal I/R injury, and the molecular mechanism of MitoQ action is closely related to the activation of the Sirt3 pathway. However, our study is still inadequate, and the specific regulatory mechanism of MitoQ on Sirt3 remains to be explored and elucidated. Collectively, our study provides new evidence for MitoQ in the treatment of renal I/R injury, and MitoQ may become a novel therapeutic agent for renal ischemic diseases.

## Data Availability

The datasets in this study are available from the corresponding authors.

## Abbreviations

AKI: Acute kidney injury
BUN: Blood urea nitrogen
Cr: Creatinine
DCFH-DA: Dichlorodihydrofluorescein diacetate
DHE: Dihydroethidium
Drp1: Dynamin-related protein 1
H/R: Hypoxia/reoxygenation
HK-2: Human renal proximal tubular epithelial cell line
I/R: Ischemia/reperfusion
Mfn2: Mitofusin-2
MMP: Mitochondrial membrane potential
ROS: Reactive oxygen species
Sirt3: Sirtuin-3
SOD1: Superoxide dismutase 1
SOD2: Superoxide dismutase 2
